# First‐Trimester Prediction of Gestational Diabetes Mellitus Based on Maternal Risk Factors

**DOI:** 10.1111/1471-0528.18110

**Published:** 2025-02-25

**Authors:** Argyro Syngelaki, Alan Wright, Cristina Gomez Fernandez, Rea Mitsigiorgi, Kypros H. Nicolaides

**Affiliations:** ^1^ Harris Birthright Research Centre for Fetal Medicine King's College London UK; ^2^ Department of Women and Children's Health, School of Life Course and Population Sciences King's College London London UK; ^3^ Institute of Health Research University of Exeter Exeter UK

**Keywords:** gestational diabetes mellitus, pyramid of pregnancy care. Risk prediction, Screening First‐trimester screening

## Abstract

**Objective:**

To develop and validate a new first‐trimester model for the prediction of gestational diabetes mellitus (GDM) based on maternal demographic characteristics and elements of medical history.

**Design:**

Prospective cohort study.

**Setting:**

Inner‐city hospital.

**Population:**

41 587 women with singleton pregnancies at 11^+0^–13^+6^ weeks' gestation, including 4231 (10.2%) who subsequently developed GDM.

**Methods:**

Logistic regression model for GDM was developed and fivefold cross‐validation was performed to assess the calibration and predictive performance of the model, assessed by the area under the receiver operating characteristic curve (AUROC) and detection rates (DRs) at different screen positive rates (SPRs).

**Main Outcome Measure:**

GDM.

**Results:**

In both parous women with a previous history of GDM and nulliparous women or parous women with no history of GDM, significant contributors to the prediction of GDM were maternal age, weight, height, ethnicity and family history of diabetes mellitus. In parous women with no previous history of GDM, there was a contribution from the birthweight z‐score of the previous pregnancy. There was good agreement between the predicted risk and observed incidence of GDM (intercept 0.000, 95% CI: −0.034, 0.034; slope 1.000, 95% CI: 0.967, 1.033). The AUROC curve was 0.757 (95% CI: 0.749, 0.765). The performance was higher for GDM treated with insulin versus metformin or diet alone. At SPR of 40%, the DR of the insulin, metformin and diet alone group was 87.2% (95% CI: 84.9, 89.3), 80.0% (77.8, 82.0) and 61.5% (59.2, 63.7), respectively.

**Conclusion:**

Assessment of risk for GDM can be achieved in the first trimester based on maternal risk factors.

## 1 Introduction

Gestational diabetes mellitus (GDM) is associated with an increased risk of maternal and perinatal short‐ and long‐term complications [1, 2, 3, 4, 5, 6, 7]. The condition is usually diagnosed at 24–28 weeks' gestation, when an oral glucose tolerance test (OGTT) is carried out. However, by this late gestational age, the fetus has already been exposed to some degree of maternal hyperglycaemia and metabolic alterations that precede it [8, 9, 10]. Diagnosis in early pregnancy and the application of preventative and therapeutic strategies could potentially reduce the incidence and impact of the disease on women and their offspring [11].

In the UK, the National Institute of Health and Care Excellence (NICE) recommends that first‐trimester OGTT is offered to women who have had GDM in a previous pregnancy and late second‐trimester OGTT to those with any one of the following risk factors: body mass index (BMI) > 30 kg/m^2^, development of GDM in a previous pregnancy, previous delivery of a macrosomic baby (≥ 4.5 kg), first‐degree relative with diabetes mellitus or racial origin with a high prevalence of diabetes such as South Asian, African‐Caribbean and Middle Eastern [12]. We have previously suggested that in screening for GDM it would be preferable to combine the various maternal factors into a multivariate logistic model, rather than treating each one as an independent screening test, as recommended by NICE [13]. The multivariate model, which was derived from the study of 75,161 singleton pregnancies, including 1827 (2.4%) that developed GDM, demonstrated the superiority of the model in the prediction of GDM compared to that of the NICE guidelines [13]. However, a major limitation of the study was that the diagnostic OGTT was not carried out in all pregnancies, but only in those with an increased random plasma glucose concentration at 24–28 weeks and consequently, the incidence of GDM in the study population was very low [13].

In the last 6 years, a more effective screening method for GDM was introduced in our hospital, and the incidence of GDM has now increased to 10%. The objectives of this study were to improve our previous prediction model using this new dataset and to examine the predictive performance of screening by the new model for different severities of GDM, reflected in whether good glycaemic control was achieved by diet and exercise alone or whether pharmacological intervention, first with the use of metformin and then with the use of insulin, became necessary.

## 2 Methods

### 2.1 Study Population

This was a prospective study in 41,587 women with singleton pregnancies attending for a routine first hospital visit at 11^+0^–13^+6^ weeks' gestation at King's College Hospital, London, between January 2018 and December 2023. At this visit, we recorded maternal demographic characteristics and medical history and performed an ultrasound scan for determination of gestational age from the measurement of the fetal crown‐rump length [14] and diagnosis of major fetal abnormalities [15]. Women gave written informed consent to take part in the study, which was approved by the NHS Research Ethics Committee (REC reference: 02‐03‐033).

The inclusion criterion for this study on screening for GDM was singleton pregnancy delivering a phenotypically normal neonate at or after 30 weeks' gestation. We excluded pregnancies with pre‐pregnancy diabetes mellitus type 1 or 2, those ending in termination, miscarriage or delivery before 30 weeks because they may not have had screening and diagnosis of GDM.

Details of maternal characteristics and the findings of the assessment at 11–13 weeks were recorded in our database. Data on pregnancy outcomes were obtained from the maternity computerised records or the general medical practitioners of the women and were also recorded in our database.

### 2.2 Maternal Characteristics and Medical History

Patients were asked to complete a questionnaire on maternal age, ethnic origin (White, Black, South Asian, East Asian or more than one ethnicity), method of conception (natural or assisted by in vitro fertilisation or use of ovulation drugs), medical history (including pre‐gestational diabetes mellitus type 1 or 2), family history of diabetes mellitus (first‐ or second‐degree) and obstetric history (parous or nulliparous with no previous pregnancies at or beyond 24 weeks). In parous women, we recorded whether any of the previous pregnancies were complicated by GDM and the gestational age at delivery and birthweight of their last baby. The questionnaire was then reviewed by a doctor together with the patient. The maternal weight and height were measured, and the body mass index was calculated in kg/m^2^.

### 2.3 Screening, Diagnosis and Management of GDM

The screening strategy for GDM in our unit is an adaptation of the NICE guidelines. In the first midwife visit, women presenting with at least one risk factor as defined by NICE guidelines are offered screening through HbA1c quantification. Those with HbA1c between 39 and 47 mmol/L are offered a 75‐g OGTT at the earliest convenience [12]; if abnormal, women are considered to have early onset GDM (when fasting sample ≥ 5.6 mmol/L and < 7 mmol/L or 2‐h sample ≥ 7.8 mmol/L and < 11 mmol/L) or pre‐gestational diabetes (if fasting sample ≥ 7 mmol/L or 2‐h sample ≥ 11 mmol/L) [12].

At 24–30 weeks' gestation, all women who did not screen positive or were not diagnosed with GDM in the first trimester are offered a timed plasma glucose 1–2 h after a standardised meal containing 50 g of carbohydrate. If the plasma glucose values are ≥ 6.7 mmol/L, women are offered a 75‐g OGTT and considered to have GDM if fasting values were ≥ 5.6 mmol/L or 2‐h values ≥ 7.8 mmol/L. Beyond 30 weeks, women are screened with a 75‐g OGTT if a large for gestational age fetus (defined as estimated fetal weight above the 90th percentile for gestational age of the Fetal Medicine Foundation fetal and neonatal growth charts [13]) or polyhydramnios is observed in routine antenatal ultrasound which is carried out at 36 weeks' gestation.

Once women are diagnosed with GDM, they are given dietary and exercise advice and encouraged to test capillary blood glucose before and 1 h after each meal. Metformin is recommended as first‐line treatment if fasting plasma glucose is ≥ 6 mmol/L or 2‐h glucose is ≥ 9 mmol/L at diagnosis or if glycaemic targets are suboptimal during a period of 1–2 weeks (pre‐meal or 1‐h post‐meal capillary glucose level ≥ 5.3 and > 7 mmol/L, respectively). Insulin is added in the event of suboptimal glycaemic control despite metformin or when metformin is not tolerated by the woman.

The medical records of the patients with GDM were reviewed to confirm the diagnosis and method of treatment.

### 2.4 Statistical Analysis

To explore maternal demographics across the outcome groups, data were expressed as means (SD) for continuous variables and *n* (%) for categorical variables. Comparison of outcome groups was by ANOVA for continuous variables and *χ* ^2^‐square test for categorical data. Distributional properties of the continuous variables were assessed graphically in order to check assumptions.

Multivariable logistic regression analysis [16] with backwards elimination was carried out to determine which of the factors among maternal characteristics and medical and obstetric history had a significant contribution in predicting GDM; covariates with *p* values > 0.05 were removed. Binned continuous variables were plotted against observed incidence to assess functional form. Maternal age in years, weight in kilograms, height in centimetres and birthweight z‐score of the last pregnancy with delivery at ≥ 24 weeks were treated as continuous variables. Maternal age at screening was centred at 35 years, maternal weight was centred at 69 kg, and maternal height was centred at 164 cm. The z‐score is the difference in standard deviations between the observed and expected birthweight for gestational age [17]. Ethnic origin, method of conception, smoking, family history of diabetes and previous GDM were treated as categorical variables. Modelling assumptions were assessed graphically. Risks for the development of GDM were obtained from this logistic regression model. Calibration of risks was assessed, and the performance of screening was evaluated by placing various cut‐offs on risks to define screen‐positive groups.

Risk calibration was assessed visually through a figure showing the observed incidence against that predicted from risk for GDM derived from the model. The plot was produced by grouping the data into bins according to risk. The observed incidence in each group was then plotted against the incidence predicted by the model (i.e., the mean risks within each group). Quantitative assessment of calibration was by recording measurements of calibration intercept and calibration slope. Calibration intercept is a measure of whether generally the risks are too high or too low. This is quantified by the estimated intercept from a logistic regression of incidence on the logit of risk with the slope fixed at 1. The intercept is a measure of the deviation of the observed incidence from the predicted. For perfectly calibrated risks, the intercept should be zero. If there is a general tendency for underestimation, so that the observed incidence is larger than that predicted, the intercept will be positive. Conversely, for overestimation, the intercept will be negative. The calibration slope assesses the calibration across the range of risks and is the slope of the regression line of the logistic regression of incidence on the logit of risk. If the risk is well calibrated, then the slope should be 1.0.

A fivefold cross‐validation study was conducted to examine the performance and calibration of the model. Essentially, the data were divided into five equal subgroups, and the model was then fitted 5 times to different combinations of four of the five subgroups and validated in the remaining fifth of the data. The performance in each of these fivefolds was summarised by risk calibration statistics, areas under the receiver operating characteristic curves (AUROC) and detection rates (DRs) for screen positive rates of 20%.

The performance of screening for the full population, both overall and in subgroups according to treatment, diet only, metformin or insulin, was assessed with DRs for fixed SPRs of 10%, 20%, 30% and 40% and by plotting the DR within each subgroup against the overall SPR.

The statistical software package R was used for data analysis.

## 3 Results

### 3.1 Screening Population

The inclusion criteria were fulfilled by 41,587 women with singleton pregnancies. There was subsequent development of GDM in 4231 (10.2%) cases, including 1816 treated with diet alone, 1487 treated with metformin and 928 treated with insulin with or without metformin. Table 1 reports trends in maternal characteristics from the unaffected group to increasing severity in GDM, reflected in the method of treatment to achieve glycaemic control. There were trends in increasing maternal age, weight and body mass index and decreasing height, a higher proportion of Black, South Asian and East Asian ethnic origins, a history of chronic hypertension, a history of a first‐ or second‐degree relative with diabetes, conceptions by assisted reproductive technologies, previous pregnancies complicated by GDM and heavier previous neonates.

**TABLE 1:** Maternal and pregnancy characteristics in the screening population.

Characteristic	Unaffected (*n* = 37 569)	GDM: diet (*n* = 1839)	GDM: metformin (*n* = 1505)	GDM: insulin (*n* = 940)	*p*
Maternal age (years)	32.6 (5.1)	33.7 (5.0)	34.0 (5.1)	34.7 (5.1)	< 0.0001
Difference in mean from unaffected	—	1.1	1.4	2.1	
Maternal weight (kg)	69.8 (14.6)	74.1 (18.1)	78.6 (18.1)	84.7 (21.1)	< 0.0001
Difference in mean from unaffected	—	4.3	8.8	14.9	
Maternal height (cm)	166.0 (6.7)	164.8 (6.8)	163.6 (6.6)	164.2 (6.9)	< 0.0001
Difference in mean from unaffected	—	−1.2	−2.4	−1.8	
Maternal BMI	25.3 (5.0)	27.2 (6.2)	29.3 (6.3)	31.3 (7.1)	< 0.0001
Difference in mean from unaffected	—	1.9	4	6	
Gestational age (days)	89.7 (3.8)	89.8 (3.8)	89.9 (3.8)	89.7 (4.0)	0.1671
Ethnicity					
White	27 084 (92.2%)	1142 (3.9%)	710 (2.4%)	435 (1.5%)	< 0.0001
Black	5851 (85.6%)	320 (4.7%)	397 (5.8%)	271 (4.0%)	
South Asian	2245 (79.4%)	187 (6.6%)	240 (8.5%)	157 (5.6%)	
East Asian	865 (78.4%)	113 (10.2%)	94 (8.5%)	32 (2.9%)	
More than one	1524 (89.1%)	77 (4.5%)	64 (3.7%)	45 (2.6%)	
Chronic hypertension					
Yes	351 (76.6%)	30 (6.6%)	37 (8.1%)	40 (8.7%)	< 0.0001
No	37 218 (89.9%)	1809 (4.4%)	1468 (3.6%)	900 (2.2%)	
Smoking					
No	36 348 (89.7%)	1789 (4.4%)	1461 (3.6%)	908 (2.2%)	0.5483
Yes	1221 (90.7%)	50 (3.7%)	44 (3.3%)	32 (2.4%)	
Family history					
None or less than 2nd degree	30 626 (91.2%)	1353 (4.0%)	1006 (3.0%)	581 (1.7%)	< 0.0001
1st degree	3845 (80.5%)	322 (6.7%)	346 (7.3%)	261 (5.5%)	
2nd degree	3098 (88.2%)	164 (4.7%)	153 (4.4%)	98 (2.8%)	
Conception					
Spontaneous	35 266 (90.0%)	1679 (4.3%)	1383 (3.5%)	849 (2.2%)	< 0.0001
In vitro fertilisation	2104 (86.1%)	149 (6.1%)	107 (4.4%)	85 (3.5%)	
Ovulation drugs	199 (86.2%)	11 (4.8%)	15 (6.5%)	6 (2.6%)	
Pregnancy history					
Nulliparous	18 762 (91.3%)	864 (4.2%)	619 (3.0%)	304 (1.5%)	< 0.0001
Parous with previous GDM	538 (44.7%)	158 (13.1%)	244 (20.3%)	265 (22.0%)	
Parous with no previous GDM	18 269 (90.9%)	817 (4.1%)	642 (3.2%)	371 (1.9%)	
Last birthweight z‐score	−0.2 (1.1)	−0.1 (1.2)	−0.1 (1.3)	0 (1.3)	< 0.0001
Difference in mean from unaffected	—	0.1	0.1	0.2	

### 3.2 Logistic Regression Model

The logistic regression model is summarised in Table 2. In this model, parous women with a previous history of GDM were treated differently from nulliparous women and parous women with no previous history of GDM. In both groups, significant contributions for the prediction of GDM were provided from weight, height, age, ethnic origin and first‐ and second‐degree family history of diabetes mellitus. In parous women with no previous history of GDM, there was a contribution from the birthweight z‐score of the previous pregnancy.

**TABLE 2:** Logistic regression analysis to determine factors defining the risk for the prediction of GDM from maternal history and characteristics. Odds ratios for continuous variables represent the odds for a unit increase in the given variable.

Term	Estimate (95% CI)	Odds ratio (95% CI)	*p*
Nulliparous: Intercept	−2.4928 (−2.5557, −2.4299)		< 0.0001
Parous, no previous GDM			
Intercept	−2.6949 (−2.7587, −2.6312)		< 0.0001
Last birthweight z	0.2162 (0.1705, 0.2618)	1.24 (1.19, 1.30)	< 0.0001
Nulliparous or parous, no previous GDM			
Maternal weight in kg – 69	0.0359 (0.0337, 0.0381)	1.0365 (1.0343, 1.0388)	< 0.0001
Maternal height in cm—164	−0.0602 (−0.0661, −0.0544)	0.9416 (0.9361, 0.9471)	< 0.0001
Maternal age in years—35	0.0599 (0.0527, 0.0672)	1.0618 (1.0541, 1.0695)	< 0.0001
Black ethnicity	0.3660 (0.2729, 0.4591)	1.4419 (1.3138, 1.5826)	< 0.0001
South Asian ethnicity	0.9245 (0.8064, 1.0426)	2.5206 (2.2399, 2.8364)	< 0.0001
East Asian ethnicity	1.2375 (1.0702, 1.4048)	3.4468 (2.9158, 4.0746)	< 0.0001
1st or 2nd degree family history	0.4455 (0.3651, 0.5259)	1.5612 (1.4407, 1.6919)	< 0.0001
Parous, previous GDM			
Intercept	−0.2608 (−0.4448, −0.0767)		0.005
Maternal weight in kg—69	0.0176 (0.0108, 0.0243)	1.0177 (1.0108, 1.0246)	< 0.0001
Maternal height in cm—164	−0.0199 (−0.0394, −0.0004)	0.9803 (0.9613, 0.9996)	0.046
Maternal age in years—35	0.0496 (0.0237, 0.0755)	1.0509 (1.0240, 1.0784)	0.0002
Black ethnicity	0.3277 (0.0238, 0.6316)	1.3878 (1.0241, 1.8806)	0.035
South Asian ethnicity	0.5292 (0.1643, 0.8941)	1.6975 (1.1786, 2.4450)	0.004
East Asian ethnicity	0.6754 (0.0553, 1.2955)	1.9648 (1.0568, 3.6529)	0.033
1st or 2nd degree family history of diabetes	0.3942 (0.1462, 0.6422)	1.4832 (1.15744, 1.9006)	0.002

**FIGURE 1:**
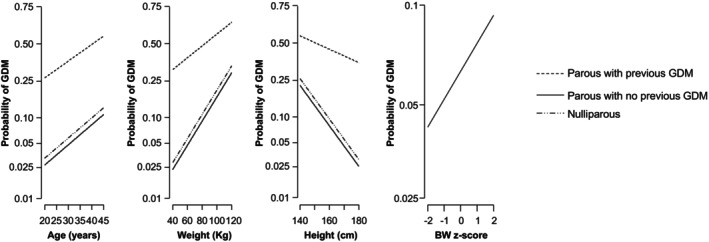
Fitted relationship between maternal age, weight, height and birthweight z‐score of previous neonate with probability of developing GDM in parous women with previous GDM (solid line), parous women without previous history of GDM (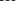 ) and nulliparous women (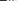 ).

**FIGURE 2:**
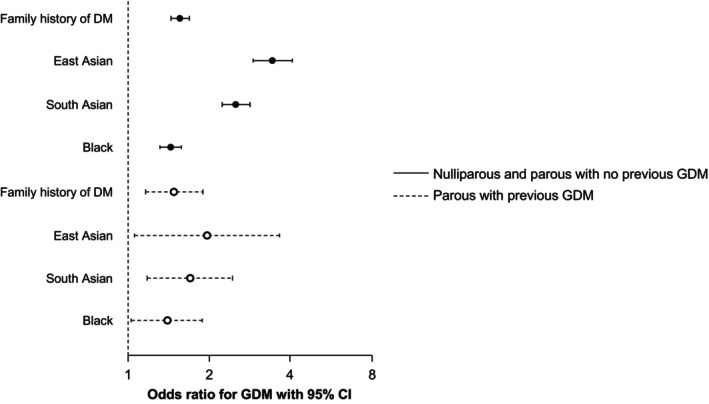
Odds ratios for development of GDM with 95% confidence intervals of various maternal characteristics for development of GDM in nulliparous women and parous women with no previous GDM (solid lines and closed circles) and parous women with previous history of GDM (interrupted lines and open circles). The reference groups are White ethnicity and no family history of diabetes.

The relationships between maternal characteristics and the probability of GDM are shown in Figures 1 and 2. The fitted probability of GDM increased with maternal age, weight and birthweight z‐score of the previous neonate and decreased with height (Figure 1). The odds ratio for GDM was increased in women with a family history of diabetes mellitus and those of East Asian, South Asian and Black ethnicity (Figure 2).

### 3.3 Performance of Screening

**FIGURE 3:**
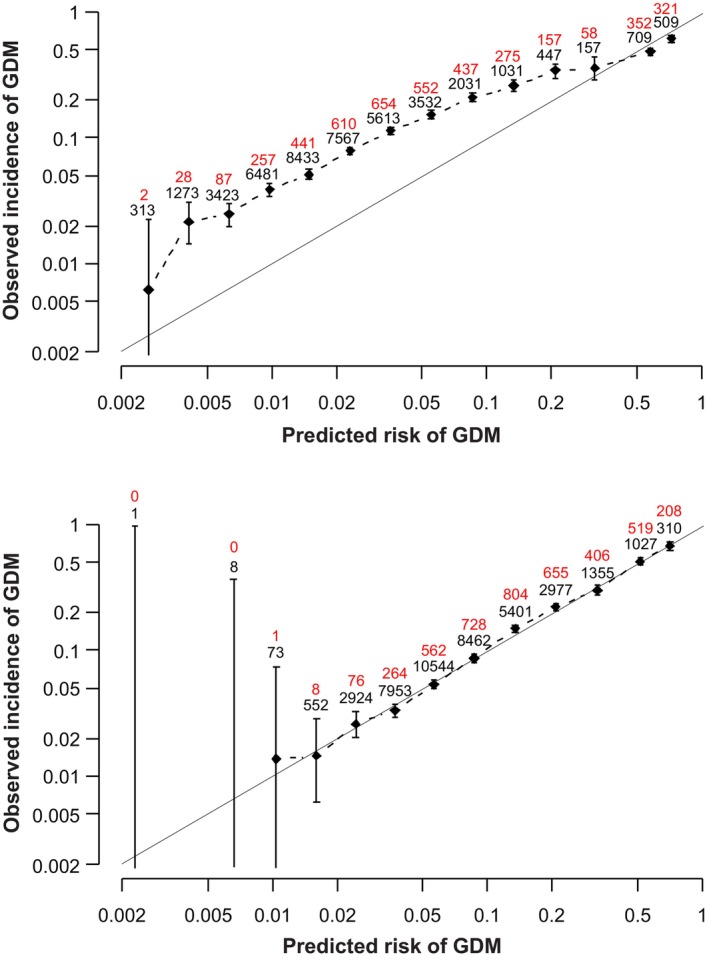
Calibration plots for screening using our previous model (top) and the new model (bottom) for prediction of GDM. The diagonal line is the line of perfect agreement between the predicted risk of GDM and observed incidence of GDM. Whiskers on datapoints are the 95% confidence interval. The numbers of women with GDM are shown in red above the total number in that predicted risk group.

The results of the fivefold cross‐validation study on the calibration intercept and slope, AUROC and DR at 20% SPR are shown in Table 3. The overall intercept and slope were 0.000 (95% CI: −0.034, 0.034) and 1.000 (95% CI: 0.967, 1.033), respectively; the AUROC was 0.757 (95% CI: 0.749, 0.765) and the DR at 20% SPR was 52.3 (95% CI: 50.8, 53.8). The calibration plots using the new and our previous model are shown in Figure 3. Using the new model, there was excellent agreement between predicted risk and observed incidence of GDM. In contrast, with the previous model, the observed incidence of GDM was considerably higher than the predicted risk [13].

**TABLE 3:** Results of the fivefold cross‐validation study on the calibration intercept and slope, area under the receiver operating characteristic curve and detection rate at a 20% screen positive rate.

Fold	Intercept		Slope		AUC (95% CI)	DR at 20% SPR (95% CI)
	Value (95% CI)	*p*	Value (95% CI)	*p*		
1	0.022 (−0.054, 0.098)	0.569	0.982 (0.908, 1.057)	0.642	0.755 (0.737, 0.772)	52.4 (49.0, 55.8)
2	0.017 (−0.059, 0.093)	0.667	1.002 (0.928, 1.075)	0.968	0.758 (0.740, 0.775)	51.9 (48.5, 55.3)
3	0.012 (−0.065, 0.088)	0.764	0.989 (0.914, 1.063)	0.768	0.756 (0.738, 0.774)	52.4 (48.9, 55.8)
4	−0.083 (−0.161, −0.005)	0.036	1.003 (0.929, 1.078)	0.933	0.759 (0.741, 0.777)	52.3 (48.8, 55.8)
5	0.031 (−0.045, 0.106)	0.426	0.999 (0.925, 1.073)	0.974	0.756 (0.739, 0.774)	51.4 (48.0, 54.8)
Overall	0.000 (−0.034, 0.034)	1	1.000 (0.967, 1.033)	1	0.757 (0.749, 0.765)	52.3 (50.8, 53.8)

**FIGURE 4:**
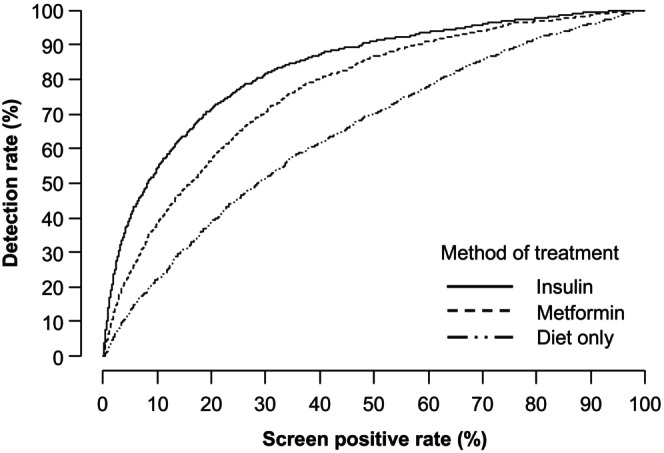
Relationship between detection and screen positive rates in screening for GDM treated by insulin (solid line) or metformin (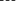 ) or diet alone (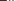 ).

Table 4 reports the predictive performance of screening for all cases of GDM and subgroups according to treatment necessary to achieve good glycaemic control, and Figure 4 plots DRs for the three treatment groups of GDM against overall SPR. The results demonstrate that the method of treatment defines the severity of the metabolic disturbance in GDM, and the DR at a given SPR is higher for GDM treated with insulin than for GDM treated with metformin or diet alone. For example, at an SPR of 40%, the DR of GDM treated with insulin was 87.2% (95% CI: 84.9, 89.3); for the metformin group, it was 80.0% (77.8, 82.0) and for those with diet alone, it was 61.5% (59.2, 63.7).

**TABLE 4:** Predictive performance of screening for all cases of GDM and subgroups according to treatment necessary to achieve good glycaemic control. Detection rate with 95% confidence interval at screen positive rates of 10%, 20%, 30% and 40%.

Screen positive rate	Gestational diabetes mellitus							
	All (*n* = 4231)		Diet alone (*n* = 1816)		Metformin (*n* = 1487)		Insulin (*n* = 928)	
	*n*	DR (95% CI)	*n*	DR (95% CI)	*n*	DR (95% CI)	*n*	DR (95% CI)
10%	1478	34.9 (33.5, 36.4)	404	22.2 (20.4, 24.2)	570	38.3 (35.9, 40.9)	504	54.3 (51.0, 57.6)
20%	2213	52.3 (50.8, 53.8)	707	38.9 (36.7, 41.2)	843	56.7 (54.1, 59.2)	663	71.4 (68.4, 74.3)
30%	2746	64.9 (63.4, 66.3)	937	51.6 (49.3, 53.9)	1052	70.7 (68.4, 73.0)	757	81.6 (78.9, 84.0)
40%	3114	73.6 (72.2, 74.9)	1116	61.5 (59.2, 63.7)	1189	80.0 (77.8, 82.0)	809	87.2 (84.9, 89.3)

## 4 Discussion

### 4.1 Main Findings of the Study

This large prospective study of women with singleton pregnancies at 11^+0^–13^+6^ weeks' gestation has demonstrated how maternal characteristics and elements of obstetric history are combined into a multivariable logistic model for early prediction of the subsequent development of GDM. We have previously reported that such a combined approach is associated with a superior predictive performance than treating each risk factor as an independent screening test, as recommended by NICE [12, 13].

In the new model, the parous women with a previous history of GDM were treated differently from nulliparous women and parous women with no previous history of GDM. In both groups, significant contributions for the prediction of GDM were provided from weight, height, age, ethnic origin, family history of diabetes mellitus and birthweight of the neonate in the last pregnancy.

The calibration plots demonstrated a very good agreement between the predicted risk for GDM derived by the new model and the observed incidence of GDM. In contrast, when our new data were examined using our previous model, the observed incidence of GDM was considerably higher than the predicted risk, reflecting the lower prevalence and higher severity of GDM in the dataset that was used to derive the previous model [13].

In the assessment of risk by the new model, the overall detection of GDM was 35%, 52%, 65% and 74% at SPRs of 10%, 20%, 30% and 40%, respectively. However, our study has demonstrated that the DR of GDM depends on the severity of the condition reflected in the method of treatment needed to achieve good glycaemic control. Thus, the DR at 40% SPR was 62%, 80% and 87% in the groups treated by diet alone, metformin and insulin, respectively, and the respective DRs at 20% SPR were 39%, 57% and 71%.

### 4.2 Strengths and Limitations

There are four main strengths of the study. First, we examined a large, unselected population of more than 40 thousand pregnant women derived from a heterogeneous inner‐city population, more than 4000 of which developed GDM. This enables precise estimation of model coefficients and performance measures as reflected in the narrow confidence intervals. Second, we asked specific questions to identify known risk factors associated with GDM and measured the weight and height of the women. Third, we used a multivariate logistic model to identify the significant factors from the maternal characteristics and to define their individual contribution in the prediction of GDM. Fourth, we examined separately the predictive performance of the model for GDM according to treatment and demonstrated the high performance in identifying the group requiring insulin treatment, which is the group at highest risk for adverse perinatal outcomes and therefore those who are likely to benefit the most from targeted intervention.

The main limitations of our study stem from the heterogeneous approach to screening, diagnosis and management of GDM in different healthcare settings. These long‐standing issues often hamper the generalisability of findings. In our study, the diagnostic OGTT was not carried out in all pregnancies, as recommended by the International Association of Diabetes and Pregnancy Study Groups [18]. It is therefore possible that some women with undiagnosed GDM might have been included in our unaffected group, with consequent underestimation of the performance of screening of our method. However, the number of such missed cases is likely to be small because our two‐step screening approach for all women at 24–28 weeks' gestation would have increased the number of women tested with OGTT. Although a plasma glucose 1–2 h after a standardised meal containing 50 g of carbohydrate is not recommended by any major guidelines or institutions, it provided a practical approach for screening for GDM at 24–28 weeks, and the decision to use a cut‐off of 6.7 mmol/L allowed for the classification of a larger proportion of women as eligible for the diagnostic OGTT.

Our management approach included the use of metformin as a first‐line pharmacological agent. Therefore, the analysis predicting GDM based on treatment status might not be reproducible in settings where different policies are applied.

Another potential limitation of the study is that the performance of screening by a model derived from the same study population was overestimated due to the overfitting of the data. Although we have addressed this issue by the fivefold cross‐validation study, it is important to externally validate our findings, not only in different populations but also using diverse existing screening strategies and diagnostic thresholds.

### 4.3 Comparison With Findings From Previous Studies

The association between maternal age, weight, height, ethnicity, family history of diabetes and previous pregnancy complicated by GDM and increasing birthweight, observed in our study, has also been highlighted in previous clinical risk prediction models for GDM [13, 19, 20, 21, 22, 23]. However, in our model, risk factors such as age, weight, height and previous birthweight were treated as continuous rather than categorical variables, whereas the previous models assumed a step function for all continuous measurements.

Some previous studies have also developed models based on logistic regression analysis and although the study populations were considerably smaller in comparison to our study, varying from 924 to 11 464 women with an incidence of GDM from 2.4% to 8.9%, they reported similar predictive performance for GDM [21, 22, 23, 24, 25, 26]. For example, Shen et al. have shown that maternal characteristics and obstetric history could predict 36% and 53% of women who subsequently developed GDM at 10% and 20% FPR, respectively [25]. The same study [25] has also highlighted the finding of this and our previous study [13] that the birthweight z‐score of a previous pregnancy should be used as a continuous variable rather than categorical limited to previous history of macrosomia or large for gestational age, because the risk for GDM increases considerably with increasing birthweight Z‐score. Another study on 3723 women, including 181 (4.9%) who developed GDM, compared the prognostic value of four previously published first‐trimester prediction models for GDM versus the traditional single risk factor approach and found that all four first‐trimester prognostic models outperform the single risk factor approach [24].

Our study has demonstrated that the predictive performance of the model depends on the severity of GDM, with substantially higher DR, at the same SPR, of GDM requiring insulin versus GDM treated by diet alone. This is particularly important because a large cohort study of 51,211 singleton pregnancies, including 2089 (4.1%) with GDM, found that in pregnancies treated with insulin versus those treated with diet alone, there was a higher rate of preterm birth, need for induction of labour, delivery by caesarean section, delivery of large‐for‐gestation neonates and admission to the neonatal unit [27]. A population‐based cohort study of 26,774 women with GDM addressed the associations of glycaemic control trajectories with adverse pregnancy outcomes and suggested that women not achieving the glycaemic targets were more likely to develop perinatal complications, and such cases were more frequently treated with insulin rather than diet or metformin only [28].

A recent review examined several studies reporting on the role of machine learning algorithms in the prediction of GDM [29]. The authors highlighted that while different machine learning models showed promise, selecting and weighing variables remain complex and it is necessary to tailor predictive models to specific populations and demographic groups. These findings highlighted the limitations of uniform guidelines for diverse populations. Moreover, studies emphasised the value of integrating clinical data into GDM prediction models.

### 4.4 Implications for Clinical Practice and Research

We advocate diagnosis and treatment of GDM as early as possible to improve maternal and neonatal outcomes, but this goal can only be achieved if all women are tested with an OGTT in both the first and third trimesters. However, such an approach may be unrealistic and impractical in the UK and many other countries. Therefore, it is important to develop a screening tool that can more effectively identify the subgroup of women who would benefit from early testing. Our screening model is not meant to replace universal GDM testing, but to enhance the selection of women for early testing. We provide DRs for different SPRs so that healthcare providers and policymakers can make decisions about which group of women should undergo earlier testing according to their resources and objectives. Further improvements in first‐trimester screening would be achieved by combining the prior risk derived from our current model with the likelihood ratio of biomarkers under investigation.

Screening and diagnosis of GDM is traditionally delayed until the late second trimester of pregnancy because the diabetogenic effects of pregnancy increase with gestation and, therefore, delayed testing maximises the DR. We have previously proposed that it would be preferable to undertake earlier testing and adjust the traditional criteria of the tests with the rationale that early identification of the high‐risk group is likely to improve pregnancy outcome because, with appropriate dietary advice and pharmacological interventions, the incidence of the disease and associated maternal and perinatal complications could potentially be reduced [30].

Our model provides an effective method of early screening for GDM, especially for severe disease requiring insulin therapy, but a prospective study is necessary to confirm our results. Additionally, the model allows the estimation of the patient‐specific a priori risk of GDM, which could be combined with potentially useful biomarkers with further improvement in the performance of screening. Numerous clinically available biomarkers have been explored for early identification of GDM, but in general, the performance has been variable [23, 31, 32, 33, 34, 35, 36, 37, 38]. A more promising approach is glycaemic profiling by continuous glucose monitoring [39, 40].

## 5 Conclusion

First‐trimester prediction of subsequent development of GDM can be achieved from a multivariable logistic model, which combines maternal characteristics and elements of obstetric history. The predictive performance of such a model is modest, but it is by far superior to the currently used NICE guidelines and it is particularly high for severe disease requiring insulin therapy.

## Author Contributions

A.S. and K.H.N. conceptualised and designed the study and wrote the first draft of the paper. A.W. carried out the statistical analysis. All authors revised and contributed to the intellectual content of the manuscript.

## Conflicts of Interest

The authors declare no conflicts of interest.

## Data Availability

Authors elect not to share the data.
